# A Pentavalent Epstein-Barr Virus-Like Particle Vaccine Elicits High Titers of Neutralizing Antibodies against Epstein-Barr Virus Infection in Immunized Rabbits

**DOI:** 10.3390/vaccines8020169

**Published:** 2020-04-06

**Authors:** Gabriela M. Escalante, Joslyn Foley, Lorraine Z. Mutsvunguma, Esther Rodriguez, David H. Mulama, Murali Muniraju, Peng Ye, Anne K. Barasa, Javier Gordon Ogembo

**Affiliations:** 1 Department of Immuno-Oncology, Irell & Manella Graduate School of Biological Sciences of City of Hope, Duarte, CA 91010, USA; gescalante@coh.org; 2 Department of Immuno-Oncology, Beckman Research Institute of City of Hope, Duarte, CA 91010, USA; jfoley@coh.org (J.F.); lmutsvunguma@coh.org (L.Z.M.); esrodriguez@coh.org (E.R.); dmulama@coh.org (D.H.M.); mmuniraju@coh.org (M.M.); pye@coh.org (P.Y.); annebarasa@uonbi.ac.ke (A.K.B.); 3 Department of Biological Sciences, Masinde Muliro University of Science and Technology, Kakamega 50100, Kenya; 4 Department of Human Pathology, University of Nairobi, Nairobi 00100, Kenya

**Keywords:** Epstein-Barr virus, glycoproteins, infectious mononucleosis, cancer, neutralizing antibody, prophylactic vaccine

## Abstract

Primary infection with Epstein-Barr virus (EBV) is associated with acute infectious mononucleosis, whereas persistent infection is associated with chronic diseases such as autoimmune diseases and various types of cancer. Indeed, approximately 2% of all new cancer cases occurring annually worldwide are EBV-associated. Currently, there is no licensed EBV prophylactic vaccine. Selection of appropriate viral protein subunits is critical for development of an effective vaccine. Although the major EBV surface glycoprotein gp350/220 (gp350) has been proposed as an important prophylactic vaccine target, attempts to develop a potent vaccine based on gp350 alone have shown limited success in the clinic. We provide data showing that five EBV glycoproteins (gp350, gB, gp42, gH, and gL) involved in viral entry and infection can successfully be incorporated on the surface of EBV-like particles (EBV-LPs). These EBV-LPs, when administered together with aluminum hydroxide and monophosphoryl lipid A as adjuvants to New Zealand white rabbits, elicited EBV glycoprotein-specific antibodies capable of neutralizing viral infection in vitro in both B cells and epithelial cells, better than soluble gp350 ectodomain. Our findings suggest that a pentavalent EBV-LP formulation might be an ideal candidate for development as a safe and immunogenic EBV vaccine.

## 1. Introduction

Epstein-Barr Virus (EBV), also known as human herpesvirus (HHV) 4, is the causative agent of infectious mononucleosis and is associated with the development of several human malignancies, including gastric carcinoma, nasopharyngeal carcinoma, Hodgkin lymphoma, and Burkitt lymphoma, among other B cell lymphoproliferative malignancies [1,2]. Approximately 200,000 cases of EBV-associated malignancies are diagnosed each year worldwide, and EBV was recently associated with the development of several autoimmune diseases [3,4,5,6]. However, despite the global health burden that EBV poses, an effective EBV prophylactic vaccine remains elusive [7].

Several prophylactic vaccine candidates have been tested in the clinic over the past three decades [8]. One consisted of a peptide from EBV nuclear antigen 3A (EBNA-3A) protein, and aimed at stimulating EBNA-3A-specific T cell responses in a Phase I clinical trial [9]. Immunization with this vaccine resulted in reduced incidence of infectious mononucleosis but not reduction of infection. The remaining vaccine candidates solely focused on the membrane glycoprotein gp350/220 (gp350), which is the major target of EBV-neutralizing antibodies (nAbs) and the most abundant glycoprotein on the surface of both EBV virions and EBV-infected cells [8]. One candidate was administered through gp350-expressing vaccinia virus; the other three were given as immunizations with recombinant gp350 in various adjuvants [10,11,12,13]. All four vaccines elicited nAb responses in Phase I/II clinical trials, but EBV infection was still detected in immunized individuals [8].

After primary infection, EBV infection is effectively controlled by the T-cell compartment, as evidenced by the presence of EBV-specific T-cells in healthy EBV+ individuals and the effectiveness of adoptive EBV-specific T-cell transfer in treating EBV-related complications in patients with both natural and induced immunosuppression [14,15,16]. Thus, T-cell mediated immunity against EBV constitutes an important correlate of immune protection against EBV reactivation and EBV-related complications. However, in the context of primary infection, the correlates of immune protection against EBV remain undefined, which has hindered the development of an effective prophylactic EBV vaccine [17]. For most licensed viral vaccines, in vitro nAb responses correlate with in vivo protection [18]. For the HHVs varicella-zoster virus and herpes simplex virus (HSV) 1, nAbs have been established as correlates of immune protection against infection [19,20,21]. Given that gp350 is the major target of nAbs during natural infection, the previous prophylactic EBV vaccine candidates focused on targeting gp350 [22]. However, although gp350 is important for facilitating viral attachment to host cells types expressing both complement receptor types 1 and 2 (CR1/CR2, also referred to as CD35/CD21), it is not essential for infection, and recombinant EBV lacking gp350 remains infectious to various permissive human cells in vitro [23]. In addition to gp350, EBV relies on four other essential glycoproteins to mediate viral fusion and entry into its main cellular targets, B cells, and epithelial cells: gB and gH/gL (the core fusion machinery) and gp42 (the tropism switch) [24,25]. In B cell infection, EBV attaches or tethers to host cells via gp350 binding to CD35/CD21 surface receptors, which triggers endocytosis of the virion [26,27,28]. This facilitates an interaction between gp42, which is complexed with gH/gL, and the HLA class II receptor, triggering the fusogenic activity of gB and allowing for fusion of the viral envelope with the endocytic vesicle membrane [24,29,30]. In epithelial cell infection, gH/gL interacts with the EphA2 receptor and a variety of integrins, triggering the fusogenic activity of gB and allowing for fusion of the viral envelope with the plasma membrane [31,32,33,34]. Interaction of EBV gp42 with gH/gL confers host cell specificity, as it promotes infection of B cells while inhibiting infection of epithelial cells by preventing binding of gH/gL to its epithelial cell receptors [30,35]. Importantly, although recombinant EBV lacking gp42 or gH does mature and egress, it does not infect either B cells (virus lacking either gH or gp42) or epithelial cells (virus lacking gH), suggesting that these glycoproteins are indeed essential for EBV infection in these two cell types [36,37].

Thus, targeting not only gp350, but also these additional glycoproteins essential for infection of both B cells and epithelial cells in a single prophylactic EBV vaccine might be key to eliciting robust nAb responses that can protect against infection in vivo. Although EBV nAbs against gH/gL, gB, and gp42 are not robustly produced during natural infection, perhaps due to masking by the immunodominant gp350, they are detectable [22,38,39]. Immunization of animals with each of the five glycoproteins results in the production of nAb responses [34,37,39,40,41,42,43,44,45]. Recently, potent nAbs against gB and gH/gL were isolated from rare memory B cells of EBV+ individuals, suggesting these antibodies are produced in humans due to natural infection, but in very low quantities [46]. Importantly, the ability of anti-gB and anti-gH/gL antibodies to neutralize infection is well-conserved in other HHVs, including cytomegalovirus (CMV) and HSV-1 and 2 [46,47,48,49,50]. Furthermore, our studies have shown that immunization of mice with UV-inactivated EBV (UV-EBV), which expresses all five glycoproteins, elicits higher titers of nAbs than immunization with any individual glycoprotein [44,51]. This information, together with our current understanding of EBV viral entry and the outcomes of previous EBV prophylactic vaccine clinical trials, suggests that to mount an effective humoral-mediated response in vivo, multiple viral antigens are required to generate a prophylactic vaccine that blocks infection of both B cells and epithelial cells, the canonical routes of primary EBV infection. 

We previously developed several virus-like particle (VLP)-based vaccine candidates incorporating either gp350, gH/gL, or gB into EBV-LPs through the Newcastle disease virus (NDV)-like particle platform, which generated moderate titers of anti-EBV nAb responses in immunized wild-type BALB/c mice [44,51]. In the present study, we used the same platform to produce, for the first time, a single pentavalent EBV-LP vaccine candidate that incorporates all five envelope glycoproteins involved in viral entry of both B cells and epithelial cells. We show that our pentavalent EBV-LPs are similar in structure, morphology, and size to EBV virions, and that they are immunogenic, eliciting glycoprotein-specific antibody responses in immunized New Zealand white rabbits, with high neutralizing activity in both B and epithelial cell lines.

## 2. Methods

### 2.1. Animals and Ethics Statement

New Zealand white rabbits were purchased from and housed at Pocono Rabbit Farm and Laboratory Inc. (PRF&L, Canadensis, PA). Animal procedures were performed in accordance with PRF&L and Beckman Research Institute of City of Hope Institutional Animal Care and Use Committee and Institutional Biosafety Committee protocols (#17093 and PRF2A). 

### 2.2. Cell Lines

Chinese hamster ovary (CHO); EBV-positive Burkitt lymphoma B cell (Raji); human embryonic kidney (HEK-293); a derivative of HEK-293 stably expressing EBNA1 protein for enhanced ability to produce recombinant proteins (HEK-293 6E); and anti-gp350 monoclonal antibody (mAb) 72A1 hybridoma producer (HB168) cell lines were obtained from American Type Culture Collection (ATCC). AGS-EBV-eGFP, a human gastric carcinoma cell line infected with recombinant Akata virus expressing enhanced green fluorescent protein (eGFP) was a kind gift of Dr. Lindsey Hutt-Fletcher (Louisiana State University Health Sciences Center, Shreveport, LA). CHO and HEK-293 cells were cultured in DMEM (ThermoFisher Scientific, Waltham, MA). HEK-293 6E cells were cultured in Freestyle F17 Expression media (ThermoFisher Scientific) without fetal bovine serum (FBS). Raji and HB168 cell lines were cultured in RPMI (ThermoFisher Scientific). AGS-EBV-eGFP was cultured in DMEM/Ham’s F-12 media (ThermoFisher Scientific) with 500 μg/mL neomycin (G418, ThermoFisher Scientific). Unless specified, all media were supplemented with 10% heat-inactivated FBS (MilliporeSigma, Burlington, MA), 1% L-glutamine (ThermoFisher Scientific), and 2% penicillin-streptomycin (ThermoFisher Scientific), and kept under general cell culture conditions (37 °C, 5% CO_2_). 

### 2.3. Antibodies

Primary mouse mAb anti-gp350 72A1 [22,52], which detects the ectodomain of gp350, was purified from HB168 cells as previously outlined [53], and used for fluorescence-activated cell sorting (FACS), immunoblot, enzyme-linked immunosorbent assay (ELISA), and neutralization assays. Alexa Fluor 488 (AF488)-conjugated goat Fab-2 anti-mouse IgG (H+L) cross-adsorbed secondary antibody (ThermoFisher Scientific) was used for FACS. Primary mouse mAbs anti-gB (CL55), anti-gp42 (F-2-1), anti-gL (E1D1), and anti-gH/gL (CL59) were a generous gift of Dr. Hutt-Fletcher and used in FACS and ELISA. Primary polyclonal rabbit anti-2A protein peptide antibody (MilliporeSigma) was used in immunoblot. Primary rabbit polyclonal anti-NDV antibody detecting nucleoprotein (NP) used in immunoblot was a kind gift of Dr. T. Morrison (University of Massachusetts Medical School [UMMS], Worcester, MA). Primary mouse polyclonal anti-gH/gL antibody raised from mice immunized with purified EBV gH/gL protein complex was used in immunoblot. Primary mouse mAb to a synthetic oligopeptide consisting of six consecutive histidine residues (6×His; clone 3D5; ThermoFisher Scientific) was used in immunoblot. Horseradish peroxidase (HRP)-conjugated secondary antibodies goat anti-mouse IgG and goat anti-rabbit IgG (Bio-Rad, Hercules, CA) were used for immunoblot and ELISA. The primary mouse mAb anti-gp350 (2L10; MilliporeSigma) was used in neutralization assays. 

### 2.4. Virus Production and Purification

eGFP-tagged Akata strain EBV (EBV-eGFP) was produced from AGS-EBV-eGFP cells as described [51]. Briefly, cells were seeded in 15-cm dishes in DMEM/Ham’s F-12 medium containing G418. After cells reached 90% confluency, media was replaced with DMEM/Ham’s F-12 medium containing 33 ng/ml 12-O-tetradecanoylphorbol-13-acetate (TPA) and 3 mM sodium butyrate (NaB) to induce lytic viral replication. Twenty-four h post-induction, media was replaced with complete DMEM/Ham’s F-12 media without G418, TPA, or NaB and cells were incubated for 4 days (37 °C, 5% CO_2_). Cell supernatant was collected, centrifuged, and filtered (0.8 µm) to remove cell debris. The filtered supernatant was ultracentrifuged using a Beckman-Coulter type 19 rotor for 70 min at 14,941 × g to pellet the virus. EBV-eGFP virus was resuspended in serum-free Opti-MEM (ThermoFisher Scientific) and titrated for infectivity in both Raji and HEK-293 cells, and stocks were immediately used for neutralization experiments or stored (−80 °C).

### 2.5. Plasmids

The pCAGGS vector [54] was modified to include a multiple cloning site (MCS) sequence, allowing for greater flexibility when adding or removing genes. Chimeric fragments of gp350 (aa 1–864), gB (aa 1–735), and gH (aa 1–679) were constructed by fusing the ectodomain of the viral proteins to the NDV-fusion (F) protein transmembrane and cytoplasmic domains. These chimeric fragments, along with native gp42 and gL, were encoded as a single transcript within the modified pCAGGS MCS vector (pCAGGS-gp350-F-gB-F-gp42-gL-gH-F). Each viral glycoprotein was separated by a short unique 2A peptide sequence (Table 1), which acts as an autocleavage signal during processing [55,56]. The construct was synthesized and sequence fidelity was verified by Genewiz (South Plainfield, NJ) via DNA Sanger sequencing. Full-length pCAGGS-NDV-M coding for NDV- matrix (M) protein and full-length pCAGGS-NDV-NP coding for NDV-NP protein have been described [57]. The construction of the pCI vector containing a puromycin resistance gene and pCAGGS vector containing the wild-type sequence for gH/gL (pCAGGS-gH/gL-WT) has been described [44,51]. 

### 2.6. Transfection of gp350-F-gB-F-gp42-gL-gH-F and Generation of Stable CHO Cell Line

5 × 10^5^. CHO cells were seeded in a 6-well plate. After 24 h, a mixture of 2 μg/well pCAGGS-EBV-gp350-F-gB-F-gp42-gL-gH-F and 0.5 μg/well pCI-puro was incubated with polyethylenimine (PEI) (1:3 DNA:PEI) in serum-free Opti-MEM (15 min, RT), then added to cells as previously described [58]. After 48 h, transfected cells were selected using 10 µg/mL puromycin selection media and cultured for three weeks. Cells were then stained with mAb 72A1 in 1×PBS (ThermoFisher Scientific) containing 1% FBS for 1 h, washed three times, stained with AF488-conjugated secondary anti-mouse IgG (30 min), and washed twice. 72A1-positive cells were sorted via FACS, seeded in 24-well plates, and allowed to recover overnight in DMEM containing 20% FBS. The following day, media was replaced with DMEM containing 10 μg/mL puromycin (ThermoFisher Scientific). This process was repeated until cells were >90% positive for stable expression of gp350. Stable cells were stained with primary antibodies specific to gp350 (72A1), gB (CL55), gp42 (F-2-1), or gH/gL (E1D1), then stained with AF488-conjugated secondary anti-mouse IgG as before and analyzed by FACS. Cells incubated with no primary antibody, primary antibody alone, secondary antibody alone, or isotype control served as controls.

### 2.7. Transfection, Generation, and Purification of EBV-LPs

CHO cells stably expressing the gp350-F-gB-F-gp42-gL-gH-F chimeric fragment were seeded in T175-cm^2^ flasks. The following day, 80–90% confluent cells were co-transfected by incubating pCAGGS-NDV-M and pCAGGS-NDV-NP plasmids (8.0 μg/flask) with PEI (1:3 DNA:PEI) in serum-free Opti-MEM (15 min), then adding the mixture to cells as previously described [58]. Supernatant from transfected cells was collected between 24–120 h post-transfection. EBV-LPs were isolated by ultracentrifugation and sucrose gradient purification as described [51].

### 2.8. SDS-PAGE, Coomassie Staining, and Immunoblotting

Purified EBV, purified EBV-LPs, cells, or purified protein were prepared for SDS-PAGE as described [59]. Prepared samples were loaded onto a 4–12% polyacrylamide gel for protein separation using 1×MES SDS running buffer (ThermoFisher Scientific). Gels were either stained with Coomassie blue or proteins were transferred from the gel to a polyvinylidene fluoride membrane using iBlot (ThermoFisher Scientific) for immunoblot. Membranes were blocked with 1% BSA in TBS (LabScientific Inc., Highlands, NJ) for 1 h, followed by incubation with specific primary antibodies against 2A, NDV-NP, gp350, gH/gL, and 6×His for 2 h and appropriate HRP-conjugated secondary antibodies for 1h after washing three times. Signal was detected with SuperSignal West Pico PLUS Chemiluminescent Substrate (ThermoFisher Scientific).

### 2.9. Transmission Electron Microcopy (TEM)

To perform morphological examination of the EBV-LPs using TEM, EBV-LPs, and EBV-eGFP virions were purified as described above, then fixed in 4% paraformaldehyde and processed as previously described [58]. 

### 2.10. Recombinant EBV Proteins

6 × His-tagged recombinant gB, gH/gL, and gp42 proteins were generous gifts of Dr. Andrew McGuire, Fred Hutchinson Cancer Center, Seattle, WA. Purified soluble gp350 ectodomain, (aa 4–863) was purchased from Immune Technology Corp, New York, NY.

Additional recombinant Fc-6 × His-tagged recombinant EBV gH/gL was produced in our laboratory. To construct gH/gL Fc-6 × His tagged plasmids, the coding sequence for gH ectodomain and full-length gL was PCR-amplified with gene-specific primers (Table 2) from pCAGGS-gH/gL-WT plasmid. The PCR product was subcloned into the Cntn1-Fc-His vector from Addgene plasmid #72065, Watertown, MA, a gift from Dr. W. Wojtowicz, Stanford University, Palo Alto, CA, and sequence fidelity verified by Sanger sequencing. 

Fc-6×His-tagged recombinant EBV gH/gL proteins were expressed by transient transfection of HEK-293 6E cells grown in Freestyle F17 Expression, FBS-free media using linear PEI transfection reagent as previously described [58]. Briefly, 200 μg of plasmid DNA and linear PEI (1:5 DNA:PEI) were added to 9 mL Opti-MEM, mixed gently, incubated for exactly 20 min at room temperature and added dropwise onto cells in a 250-mL Erlenmeyer flask. Culture media was harvested six days post-transfection by centrifugation and filtration through a 0.22-µM filter. The Fc-6×His-tagged EBV proteins in the media were purified using Protein A spin columns (Takara Bio Inc., Kusatsu, Japan), buffer-exchanged and concentrated into 1×PBS using Amicon Ultra 15 centrifugal filter units (MilliporeSigma) and quantified via nanodrop.

### 2.11. Immunization of Rabbits

The 10–12-week-old female and male wild-type New Zealand white rabbits were divided into four groups (n=6) and immunized subcutaneously as follows: Group 1) 50 µg of EBV-LPs, to determine their efficacy as a prophylactic vaccine; Group 2) 50 µg of UV-inactivated EBV (UV-EBV) as a positive control; Group 3) 25 µg soluble gp350 ectodomain (4-863 amino acids) as an additional positive control; Group 4) TNE buffer alone as a negative control. All immunizations were adjuvanted with 500 μg aluminum hydroxide (alum) mixed with 50 µg monophosphoryl lipid A from *Salmonella enterica* serotype minnesota Re 595 (MPL) in TNE buffer. Total protein of EBV-LPs and UV-EBV was quantified using Micro BCA™ Protein Assay Kit (ThermoFisher). Rabbits were immunized (Day 0) and then boosted twice (Day 28 and 42). Rabbits were bled 7 days prior to the start of the immunization regimen and on post-immunization Days 14, 35, 49 and 70, then humanely euthanized for terminal bleeding on Day 90.

### 2.12. Determination of Glycoprotein-Specific Antibody Titers in Serum of Immunized Rabbits

The IgG titers were measured by ELISA as described using soluble gp350, gB, gp42 and gH/gL as target antigens [51,58]. First, 96-well microtiter plates (Nunc-Immuno Plate Maxisorp) were coated with 25 ng/well of the target antigen in 1×PBS (pH 6.2) at 4 °C overnight and blocked with 0.1% Tween in PBS + 1% BSA. Equal amounts of sera from each animal for each treatment group and timepoint were pooled and serially diluted in 1×PBS (1:100, 1:300, 1:900, 1:27,000, and 1:81,000), added to the plate in quadruplicates, and incubated for 2 h at RT. The plate was washed 3 times and then incubated with HRP-labeled anti-rabbit secondary antibody at RT for 1h. Plates were washed three times and the substrate ABTS (Sera Care) was added. The reactions were stopped with ABTS stop solution (Sera Care). To determine antibody titer, optical density (OD) was read at 405 nm with a spectrophotometer (Filermax® F3, Molecular Devices). The assay was independently repeated two times with either individual animal serum or pooled sera.

### 2.13. Purification of IgG Antibodies from Rabbit Sera, and Determination of Glycoprotein-Specific Antibody Titers

Equal amounts of Day 70 sera from each of the treatment groups was pooled. Pooled sera were then diluted 1:10 in equilibration/binding/wash buffer (1.5 M glycine, 3 M NaCl, pH 9.0) and purified through protein A spin columns (Takara Bio Inc.) following the manufacturer’s protocol. Purified IgG concentrations for each group were determined via nanodrop. To determine titers of anti-gp350, anti-gB, anti-gp42, and anti-gHgL antibodies in purified IgGs from each group, purified IgGs were serially diluted in 1×PBS (to 25, 12.5, 6.25, 3.125, and 1.56 µg/mL) and used as a primary antibody for ELISA, performed as described above.

### 2.14. EBV-eGFP Neutralization Assay in B cells and Epithelial Cells

In vitro neutralization was performed using human B cell (Raji) or epithelial (HEK-293) cell lines as described [38]. To conduct neutralization, we first titered EBV-eGFP in individual cell types. Briefly, each individual cell line was seeded overnight at a density of 5 × 10^4^ in quadruplicate in 48-well-plates. The individual cell lines were then incubated with 5, 10, 20, 30, and 50 µL of the purified virus in a total volume of 100 µL of virus plus serum-free media for 48 h at 37 °C. Infected cells (eGFP-positive) were quantified by FACS by acquiring a total of 10,000 events and analyzed using FlowJo Software (FlowJo LLC, Ashland, Oregon) as described [59]. 

Upon determination of virus titer, cells were seeded as above. Purified IgGs and neutralization control mAbs 72A1 (positive control) and 2L10 (negative control) were serially diluted in serum-free media to 50, 25, 12.5, 6.25, 3.125, and 1.56 µg/mL and incubated for 1h at 37 °C with a known quantity of the virus that yielded 40–70% infectivity in the respective cell type used. The mixtures of purified IgGs and virus were then added to the seeded cells and incubated for 2 h at 37 °C. Infected cells were washed three times in 1× PBS to remove excess IgGs and circulating viruses, and complete media was added, and cells incubated for 48 h at 37 °C. The number of eGFP-positive cells was enumerated using FACS by acquiring 10,000 events in each case. All dilutions were performed in quadruplicate and assays repeated at least once. 

### 2.15. Statistical Analysis

Statistical analyses were performed using GraphPad Prism 8 Software (GraphPad Software, San Diego, CA). Multiple comparisons between groups was calculated using One-way ANOVA; differences between two groups were calculated using Tukey’s post hoc test. IC_50_ values were calculated based on neutralization of EBV-eGFP by purified IgGs in Raji and HEK-293 cells using nonlinear, dose-response regression analysis.

## 3. Results

### 3.1. Construction, Purification, and Characterization of EBV-LP Vaccine Candidate That Incorporates Five EBV Glycoproteins

**Figure 1:**
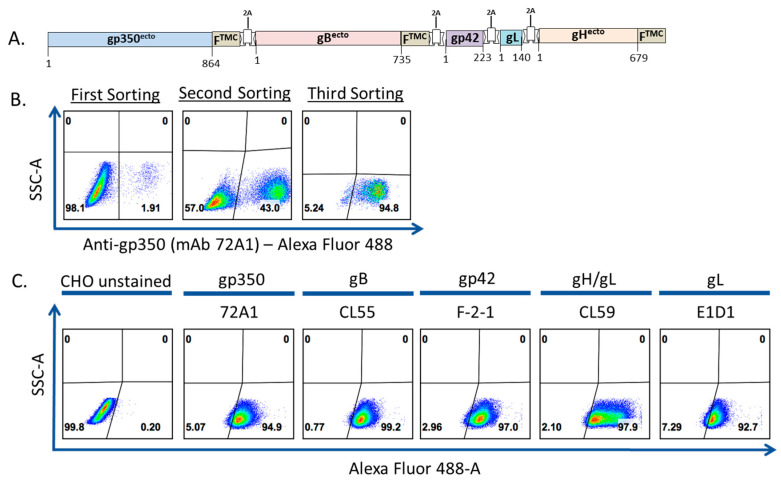
Generation of a Chinese hamster ovary (CHO) cell line stably expressing five recombinant Epstein-Barr virus (EBV) glycoproteins. (**a**) Schematic of the chimeric glycoprotein construct sequence inserted into a modified pCAGGS vector for expression in CHO cells. The construct consists of the five EBV glycoproteins indicated, interspersed with unique 2A autocleavable linker sequences. The ectodomains of gp350, gB, and gH are fused to the transmembrane/cytoplasmic (TMC) domain of Newcastle disease virus-F (NDV-F) protein to facilitate incorporation onto the virus-like particle (VLP) surface. Amino acid numbers in each glycoprotein covered by the construct are shown; (**b**). Sequential enrichment of CHO cells expressing five EBV glycoproteins. CHO cells were co-transfected with pCAGGS-EBV-gp350-F-gB-F-gp42-gL-gH-F and pCI-Puro plasmids. Forty-eight h post-transfection, transfected cells were selected using 10 µg/mL puromycin selection media. Selected cells were stained with anti-gp350 mAb 72A1, followed by staining with AF488-conjugated secondary anti-mouse IgG, and sorted via FACS (first sorting). The collected cells were maintained in 10 µg/mL puromycin, and the sorting process was repeated twice (second and third sorting) until >90% cells were positive for gp350 expression; (**c**) Fluorescence-activated cell sorting (FACS) analysis of stable CHO cells expressing five EBV glycoproteins. To confirm stable expression of all glycoproteins in the gp350-positive CHO cells, cells were additionally stained with anti-gB mAb (CL55), anti-gp42 mAb (F-2-1), anti-gH/gL mAb (CL59), or anti-gL mAb (E1D1) followed by staining with AF488-conjugated secondary anti-mouse IgG and analyzed by FACS. The transfected cells were compared to unstained CHO (shown), CHO stained with primary antibody alone, CHO stained with secondary antibody alone, or CHO stained with isotype control.

To produce a pentavalent EBV-LP incorporating glycoproteins involved in viral entry of B cells and epithelial cells, we first generated CHO cells stably expressing gp350, gB, gp42, and gH/gL. We replaced the transmembrane domains of gp350, gB, and gH with the transmembrane/cytoplasmic domain of the NDV-F protein to allow glycoprotein incorporation into EBV-LPs [57]. We assembled these DNA sequences, along with the sequences for native gp42 and gL, together as a single chimeric polycistronic construct, which we cloned into a pCAGGS-MCS mammalian expression vector, separated by distinct 2A peptide autocleavable sequences (Table 1), generating the pCAGGS-EBV-gp350-F-gB-F-gp42-gL-gH-F vector (Figure 1a) [60]. We then co-transfected CHO cells with this vector and a pCI-puro plasmid, and we cultured the transfected CHO cells in DMEM supplemented with 10 μg/mL puromycin. To enrich puromycin-selected stable gp350-F-gB-F-gp42-gL-gH-F cells, we stained the cells with anti-gp350 primary antibody (72A1), followed by staining with AF488-conjugated secondary goat anti-mouse IgG, then sorted stained cells via FACS and further cultured sorted cells in puromycin selection media. The sorting process was repeated two times and the gp350-positive cell population increased progressively with each sort, from 1.9% to 43.0% to 94.8% (Figure 1b). After multiple passages and continuous culture in 10 μg/mL puromycin culture media, we stained the cells with primary antibodies specific to each of the five glycoproteins and determined the cell population to be >90% positive for all glycoproteins by FACS (Figure 1c).

**Figure 2:**
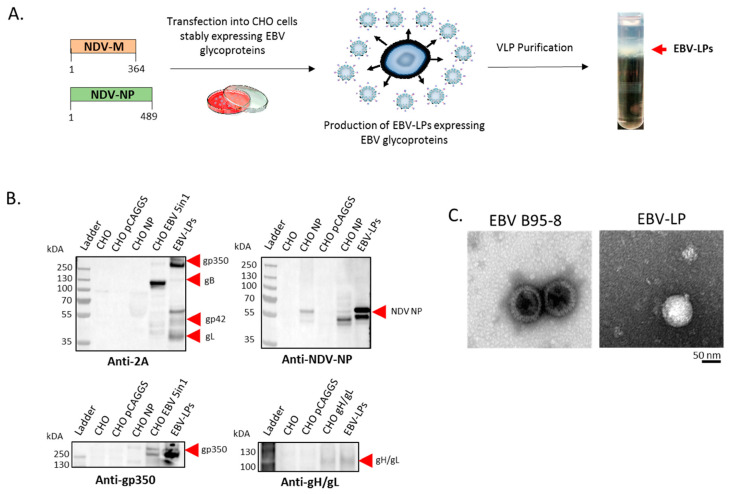
Production and characterization of EBV-like particles (EBV-LPs). (**a**) Generation of EBV-LPs in CHO cells stably expressing five EBV glycoproteins. CHO cells stably expressing the five glycoproteins were co-transfected with plasmids encoding NDV-M and NDV-NP proteins (amino acid numbers in each of the NDV proteins are shown) to induce production of EBV-LPs. After transfection, supernatants were collected between 24–120 h post-transfection and EBV-LPs were pelleted by ultracentrifugation and purified through a sucrose density gradient (EBV-LP layer shown by red arrow); (**b**) Immunoblot analysis of purified EBV-LPs. After lysis, purified EBV-LPs were resolved on a 4–12% polyacrylamide gel, transferred to a polyvinylidene fluoride membrane, and analyzed by immunoblot with anti-2A polyclonal, anti-NDV-NP polyclonal, anti-gp350 monoclonal (72A1), and anti-gH/gL polyclonal primary antibodies, as indicated. Untransfected CHO cells (CHO), CHO cells transfected with “empty” pCAGGS vector (CHO pCAGGS), CHO cells transfected with pCAGGS-NDV-NP vector alone (CHO NP), CHO cells transfected with pCAGGS-gH/gL-WT (CHO gH/gL), and stable CHO cells expressing EBV gp35-F-gB-F-gp42-gL-gH-F (CHO EBV 5in1) served as controls when indicated; (**c**) TEM analysis of purified EBV-LPs. Purified EBV virions and EBV-LPs were fixed in 4% paraformaldehyde and adsorbed to glow-discharged, carbon-coated, 200-mesh EM grids. Micrographs were collected using an FEI Tecnai 12 TEM and recorded with a Gatan 2 × 2 k CCD camera at a magnification of 21,000X and a defocus value of ∼1.5 μm.

To produce EBV-LPs, we co-transfected stable CHO cells expressing all five EBV glycoproteins with pCAGGS-NDV-M and pCAGGS-NDV-NP plasmids (Figure 2a). We collected supernatant containing EBV-LPs between 24–120 h post-transfection, purified EBV-LPs from the supernatant on a sucrose gradient (Figure 2a), resuspended purified EBV-LPs in TNE buffer, and characterized their structure and composition. We confirmed expression of each of the five glycoproteins using immunoblot (Figure 2b). We used primary rabbit polyclonal anti-2A antibody to detect the 2A signal peptide attached to the end of gp350, gB, gp42, and gL in purified EBV-LPs (Figure 2b); however, gH was not detected via 2A due to the cleavage pattern in the construct (Figure 1a). We used additional antigen-specific mouse primary antibodies against gp350 and gH/gL to successfully detect these glycoproteins in purified EBV-LPs (Figure 2b). We also used primary rabbit polyclonal anti-NDV-NP antibody to detect NDV-NP protein in the purified EBV-LPs (Figure 2b). We used TEM for structural characterization and confirmed that the size and morphology of EBV-LPs was comparable to that of purified EBV virions (Figure 2c).

### 3.2. Pentavalent EBV-LPs Are Immunogenic and Elicit EBV-Glycoprotein-Specific Antibodies in Rabbits

**Figure 3:**
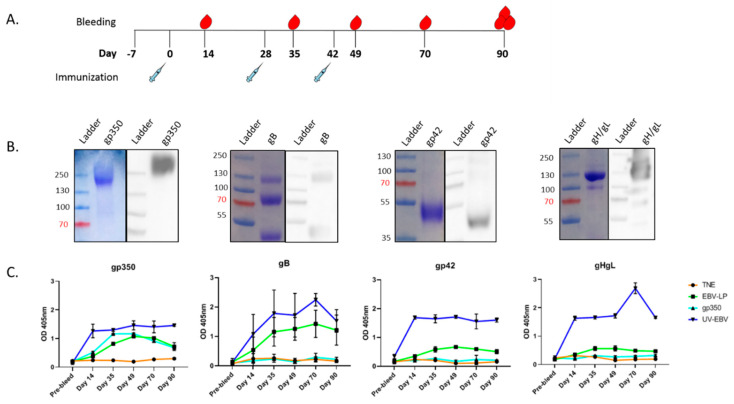
Antibody response in EBV-LP-immunized New Zealand white rabbits. (**a**) New Zealand white rabbit immunization and bleeding schedule schematic. Rabbits were immunized and bled as detailed in the Materials and Methods; (**b**) Biochemical characterization of recombinant EBV proteins used as ELISA target antigens. Coomassie stain (left) and immunoblot (right; performed using anti-6×His primary antibody) of soluble gp350 ectodomain, and recombinant EBV gB, gp42, and gH/gL proteins, which were used as target antigens in ELISA assay in panel C; (**c**) EBV-specific antibody responses in sera. IgG titers in immunized animals were measured by ELISA for each glycoprotein; proteins described in panel B were used as target antigens at 25 ng/well, and sera from immunized rabbits for each treatment group and timepoint were pooled, serially diluted, and used as primary antibody (1:100 dilution shown). Primary mouse mAbs anti-gp350 (72A1), anti-gB (CL55), anti-gp42 (F-2-1), anti-gL (E1D1), and anti-gH/gL (CL59) were used as positive controls where appropriate (not shown). Antibody binding was detected with HRP-labeled anti-rabbit IgG secondary antibody, and optical density (OD) was read at 405 nm with a spectrophotometer. ELISA assay was performed for each sample in quadruplicate, and results are expressed as mean ± standard deviations (SD). The assay was independently repeated two times with either individual animal serum or pooled sera.

To determine EBV-LP immunogenicity, we immunized New Zealand white rabbits (n=6/treatment group) subcutaneously on Day 0 (primary immunization) and Days 28 and 42 (boosts), with 50 µg EBV-LPs suspended in TNE buffer adsorbed to 500 µg alum and 50 µg MPL (Figure 3a). UV-EBV (50 µg) and soluble gp350 ectodomain (25 µg) served as positive controls; TNE buffer served as a negative control. To evaluate antibody response over time, we collected serum from individual rabbits before immunization (pre-bleed) and on post-immunization Days 14, 35, 49, 70, and 90 (terminal bleed) (Figure 3a). We observed no signs of local or systemic inflammation or changes in any rabbit behavior during the study.

To evaluate the production of antibodies specific to gp350, gB, gp42, and gH/gL in response to EBV-LP immunization, we performed ELISA. We used recombinant EBV glycoproteins as the target antigens, after confirming their integrity via Coomassie blue staining and immunoblot (Figure 3b). We pooled individual rabbit serum from each treatment group and timepoint, then diluted pooled sera for use as the primary capture antibody for the assay (Figure 3c). Although the increase in titers of gH/gL and gp42-specific antibodies in EBV-LP-treated animals was not statistically significant compared to the TNE-negative control group, we observed specific antibody responses for each of the glycoproteins expressed on the EBV-LPs, which peaked between Days 49 and 70 (Figure 3c). The quantity of detected antibodies increased with booster immunizations for all glycoproteins except gH/gL. Since immunization with EBV-LPs resulted in a significantly lower IgG titer than UV-EBV for all five glycoproteins tested, this suggests that natural virions are more immunogenic than EBV-LPs. As expected, for rabbits immunized with soluble gp350 ectodomain, only anti-gp350 antibodies were detected. No antibodies were detected in pre-immunization sera or sera from TNE-treated rabbits. These antibody response data corroborate that we successfully packaged—for the first time—five EBV surface glycoproteins on a VLP, and that these EBV-LPs are immunogenic.

### 3.3. EBV-LPs Produce Robust nAb Responses in Immunized Rabbits That Prevent EBV Infection of Both B Cell and Epithelial Cell Lines

**Figure 4:**
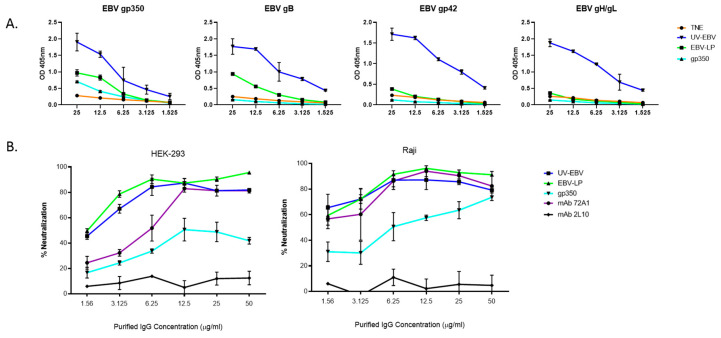
In vitro neutralizing activity of purified IgGs from rabbits immunized with EBV-LPs. (**a**) Titration of purified Day 70 IgGs specific to EBV. Equal amounts of Day 70 sera from immunized rabbits from each treatment group (UV-EBV, EBV-LP, gp350, and TNE) were pooled, and total IgG antibodies were purified via protein A spin columns. Total IgGs were serially diluted (25, 12.5, 6.25, 3.125, and 1.56 µg/mL) and EBV-glycoprotein-specific IgG titers were determined in each dilution in quadruplicate by ELISA as described in Figure 3C. Results are expressed as mean ± SD; (**b**) EBV-eGFP neutralization assay in Raji B cells and HEK-293 epithelial cells. Neutralization activity of purified Day 70 IgGs was determined by incubating known quantities of EBV-eGFP that result in 40–70% infectivity with serially diluted purified IgGs (50, 25, 12.5, 6.25, 3.125, and 1.56 µg/mL µg/mL) from all treatment groups for 1 h at 37 °C. The mixtures of purified IgGs and virus were then added to previously seeded cells and incubated for 2 h at 37 °C, after which the cells were thoroughly washed three times with 1×PBS and given complete media. Cells were collected after 48 h and infected cells (eGFP-positive) were quantified using FACS. Cells incubated with virus or media alone served as positive and negative controls for infection, respectively, and resulting infectivity was used to calculate % neutralization. Neutralizing anti-gp350 mAb 72A1 and non-neutralizing anti-gp350 mAb 2L10 served as positive and negative controls for neutralization, respectively. Results were normalized to the TNE group and are shown as mean ± SD.

To determine neutralization activity, we pooled Day 70 sera for each group, including controls, then column-purified sera to isolate total IgGs. We quantified purified IgG titers (Figure 4a), then incubated varying concentrations of IgGs (1.56, 3.125, 6.25, 12.5, and 25 µg/mL) with EBV-eGFP for 1 h, before adding them to 5 × 10^4^ cultured B cells (Raji) or epithelial cells (HEK-293) to determine nAb activity. Neutralizing anti-gp350 mAb 72A1 and non-neutralizing anti-gp350 mAb 2L10 were used as positive and negative controls for neutralization, respectively.

We measured nAb activity using FACS to quantitate eGFP-positive cells, and normalized results to the TNE-negative control group. EBV-eGFP pre-incubated with mAb 72A1 or pooled IgGs from rabbits immunized with UV-EBV, EBV-LPs, or soluble gp350 ectodomain resulted in a dose-dependent increase of EBV neutralization (Figure 4b). As expected, mAb 2L10 did not neutralize EBV infection. nAb titers against UV-EBV- or EBV-LP-immunized rabbits were significantly higher than titers against soluble gp350 ectodomain alone in both B cells (UV-EBV vs. gp350 p-value<0.05 for 3.125, 6.25, 12.5, and 25ug/mL; EBV-LP vs. gp350 p-value<0.05 for 12.5ug/mL and above) and epithelial cells (UV-EBV and EBV-LP vs. gp350 p-value<0.05 for all concentrations), neutralizing ~50–90% of the virus in all IgG concentrations tested, compared to ~20–70% for nAb titers against gp350 alone. This encouraging finding suggests that a multivalent EBV-LP vaccine provides better protection against EBV than gp350 alone. However, whether all five glycoproteins contribute to the observed neutralizing activity or whether this neutralizing activity can be replicated in vivo remains to be tested.

## 4. Discussion

Previous prophylactic EBV vaccines that focused on the immunodominant glycoprotein gp350, the main target of naturally occurring EBV nAbs, failed to reduce rates of infection in the clinic [10,11,12,13]. Since EBV utilizes additional essential glycoproteins to mediate infection, which are also targets of EBV nAbs [23], a successful prophylactic EBV vaccine likely requires inclusion of multiple antigens important for EBV infection. In this study, we generated a pentavalent vaccine candidate against EBV incorporating five key glycoproteins involved in mediating viral entry in B cells and epithelial cells—gp350, gB, gp42, and gH/gL—into a single EBV-LP. Purified EBV-LPs resembled purified EBV virions in size, shape, and morphology. Our newly generated EBV-LPs were immunogenic, eliciting IgG antibody responses specific to all five glycoproteins in immunized rabbits, with anti-gp350 and anti-gB IgG titers being the highest while gp42 and gH/gL IgG titers being elicited at lower levels. Importantly, purified IgGs from immunized animals were capable of neutralizing EBV infection in EBV-permissive B cell and epithelial cell lines. To the best of our knowledge, this is the first pentavalent VLP-based EBV vaccine candidate.

Our analysis of antibody kinetics showed that antibody titers against all five glycoproteins peaked between Day 49 and Day 70. However, although both UV-EBV and EBV-LP groups followed a similar IgG titer pattern, sera from UV-EBV-immunized rabbits maintained higher glycoprotein-specific IgG titers overall than sera from EBV-LP-immunized rabbits, for all time points tested. Incubation of purified IgGs from UV-EBV or EBV-LP-immunized rabbits with EBV-eGFP reduced infection in a dose-dependent manner in both cell types, neutralizing up to ~90% of infection at the highest IgG concentration tested. Surprisingly, neutralization activity of purified IgGs from EBV-LP-immunized rabbits was comparable to that of sera from UV-EBV-immunized rabbits on Day 70, despite the sera from UV-EBV-immunized rabbits displaying higher total glycoprotein-specific IgG titers. Purified IgGs from EBV-LP-immunized animals showed better neutralizing activity than either purified IgGs from gp350-immunized animals or purified nAb 72A1. This translated into an IC_50_ of <4 µg/mL in both B cells and epithelial cells (Table 3), which is comparable to previously published neutralizing antibody IC_50_ values for CMV [47], Zika virus [61], and *Plasmodium falciparum* [62]. Given the levels of glycoprotein-specific purified IgGs, we speculate that most of the observed neutralizing activity originated from gp350- and gB-specific IgGs. gp350 is a known source of nAbs, particularly for B cells, and antibodies against gB have been shown to neutralize infection of both B cells and epithelial cells [39,42,46]. Thus, our results support previous studies. Nevertheless, we cannot rule out the presence of potent gH/gL- and gp42-specific nAbs, albeit at low titers, and we are currently investigating the role of each EBV-LP glycoprotein in nAb generation in an independent study. 

Our encouraging findings support the use of a pentavalent EBV vaccine targeting key glycoproteins involved in infection of both B cells and epithelial cells. However, these observations correspond to a time point at which total IgG titers peaked. The durability and overall titers of glycoprotein-specific IgGs in EBV-LP-immunized rabbits before and beyond Day 70 were inferior to IgGs in UV-EBV-immunized rabbits, a control which we have consistently included in our previous studies as a “gold standard” comparator [44,51]. Furthermore, the levels of gp42- and gH/gL-specific IgG titers in EBV-LP immunized rabbits were particularly low when compared to gp350- and gB-specific IgGs. This low level could be the result of poor protein expression due to using a polycistronic expression platform under a single promoter [60], or suggest potential antigen competition. Given the pattern observed in glycoprotein-specific IgG titers (anti-gp350 ≈ anti-gB > anti-gp42 > anti-gH/gL), which follows the order in which the glycoprotein genes are arranged in the polycistronic expression construct after the promoter, we believe the reason for low anti-gp42 and anti-gH/gL IgGs to be the former. As it has been previously reported for the 2A multi-expression system, it is possible that the farther from the promoter, the lower the gene expression, resulting in unequal glycoprotein expression and incorporation into EBV-LPs [60]. This seemingly contradicts our results showing that >90% of cells in the stable CHO cell population expressed all five glycoproteins; it is possible that although all glycoproteins are expressed, they are not expressed equally in density, resulting in varying levels of glycoproteins displayed on the CHO cell surface and thus unequal incorporation into EBV-LPs. Breaking the polycistronic construct into two separate expression constructs (gp350-gB and gp42-gH/gL) with distinct promoters in a bidirectional vector might improve glycoprotein expression and immunogenicity.

## 5. Conclusions

Our VLP system also faces challenges regarding production efficiency and scalability, similar to previous vaccine platforms [63,64]. Mammalian cells, the only platform for achieving ideal protein folding and post-translational modifications for faithful VLP assembly, suffer from reduced productivity compared to other VLP systems [65]. In addition, achieving efficient transfection while maintaining cell viability is necessary for successful VLP production; this requires a large number of cells to produce relatively limited amounts of VLPs. Thus, in its current state, our system is hindered by low productivity relative to the number of cells needed to produce a small working VLP stock for animal experiments, with no potential for scalability. To boost overall immunogenicity, establish efficient and robust expression of all glycoproteins, and improve efficiency and scalability of the EBV-LP production process, future studies will explore the use of live-attenuated vectors, such as modified vaccinia Ankara (MVA), chimpanzee adenovirus 3, and/or vesicular stomatitis virus to deliver EBV-LPs and EBV antigens, as was done recently for Ebola virus with very promising results in pre-clinical and Phase Ib clinical trials [66,67,68,69,70,71,72,73]. In the field of EBV vaccinology, viral vaccine vectors, particularly adenoviral and MVA vectors, have been used both pre-clinically and clinically in the context of therapeutic and prophylactic vaccination. For example, an early preclinical study used an adenoviral vector expressing epitopes from the latent proteins LMP1 and LMP2 that successfully reduced tumor burden and increased survival in mice bearing LMP1-expressing tumors [74]. An MVA vector expressing the latent proteins EBNA1 and LMP2 was successful in eliciting EBV-specific cellular responses in nasopharyngeal carcinoma patients in two clinical trials [75,76]. More recently, a heterologous prime-boost vaccination strategy using both adenoviral and MVA vectors expressing EBNA1, succeeded in both protecting against and treating EBNA1+ lymphoma in a mouse model [77]. By adopting similar strategies in a prophylactic context, we hope to improve our current platform and build on our promising EBV-LP prophylactic vaccine candidate.

## Figures and Tables

**Table 1:** 2A protein sequences used in expressing polycistronic EBV glycoprotein transcript.

2A Position	Nucleotide Sequence	Amino Acid Sequence
gp350-gB	GCTACTAACTTCAGCCTGCTGAAGCAGGCTGGAGACGTGGAGGAGAACCCTGGACC	A T N F S L L K Q A G D V E E N P G P
gB-g42	GCCACCAATTTCTCGTTACTTAAACAAGCGGGTGACGTTGAAGAGAATCCGGGACCT	A T N F S L L K Q A G D V E E N P G P
g42-gL	GCGACTAACTTCTCATTGTTGAAACAGGCAGGAGATGTCGAAGAGAACCCTGGTCCA	A T N F S L L K Q A G D V E E N P G P
gL-gH	GCAACGAATTTCTCCCTTCTAAAGCAAGCCGGTGACGTGGAGGAGAATCCCGGACCC	A T N F S L L K Q A G D V E E N P G P

**Table 2:** Primers used to clone and express recombinant Fc-6x His-tagged EBV gH/gL.

Primer Name	Primer Sequence (5’-3’)
*Cloning Primers*	
EBV gH-gL-Fc-His FWD	AAAAAGCGGCCGCGCCACCATGCGTGCTGTTGGTGTATTTC
EBV gH-gL-Fc-His REV	AAAAAACTAGTGTGTGCTCTTTCTTCATACAGG
*Sequencing primers*	
FC-Hisseqprimer4	GCTTTAATAAGATCTCTAG
FC-Hisseqprimer5	TGCTGGGCACGGTGGGCATG
FC-Hisseqprimer6	GGGTCTTTTCTGCAGAAGCTTG

**Table 3:** EBV neutralization IC_50_ values for purified IgGs from gp350, UV-EBV and EBV-LP -treated rabbits, and for anti-gp350 mAbs 72A1 and 2L10.

	IC_50_ *(µg/mL)				
Cell-Line	gp350	UV-EBV	EBV-LPs	mAb 72A1	mAb 2L10
HEK-293	5.67	3.11	2.85	6.25	nd
Raji	8.97	3.42	3.71	4.81	nd
